# Simultaneous bicompartmental bucket-handle meniscal tears with intact anterior cruciate ligament: a case report

**DOI:** 10.1186/1752-1947-4-34

**Published:** 2010-02-01

**Authors:** Marios G Lykissas, George I Mataliotakis, Nikolaos Paschos, Christos Panovrakos, Alexandros E Beris, Christos D Papageorgiou

**Affiliations:** 1 Department of Orthopaedic Surgery, University of Ioannina, School of Medicine, Ioannina, 45110, Greece

## Abstract

**Introduction:**

Bucket handle tear of the menisci is a common type of lesion resulting from injury to the knee joint. Bucket handle injury of both menisci in almost all cases is associated with a lesion to either the anterior or the posterior cruciate ligament of the knee joint. We describe a case of acute bucket-handle tear of the medial and lateral menisci with intact anterior and posterior cruciate ligaments in a dancer. To the best of our knowledge, there are no previous reports of this type of injury in the literature.

**Case presentation:**

A 28-year-old Caucasian Greek woman presented to the emergency department after sustaining an injury to her right knee during dancing. An MRI evaluation demonstrated tears in both menisci of the right knee, while the anterior and posterior cruciate ligaments were found to be intact. A partial medial and lateral meniscectomy was then performed. At a follow-up examination six months after her injury, clinical tests demonstrated that our patient's right knee was stable, had a full range of motion and had no tenderness. She was satisfied with the outcome of the operation and returned to her pre-injury activities.

**Conclusion:**

We present the first case in the literature that describes a combined bucket-handle injury of both the medial and lateral menisci with an intact anterior cruciate ligament. The clinical examination of the anterior cruciate ligament was unremarkable, with no signs of deficiency or rupture. The posterior cruciate ligament was also intact. On magnetic resonance imaging, the ligaments were visualised as intact in all their length. These findings were confirmed by arthroscopic evaluation.

## Introduction

The erect position of the human body requires special structures to support its weight. The menisci have an important role in joint stability and in load transmission across the knee joint [1,2]. There are different types of meniscal tears (longitudinal, bucket-handle, horizontal, radial and oblique), and each of them has different characteristics depending on the mechanism of the injury and the location [3].

Bucket-handle tears represent approximately 10% of all reported cases of meniscal tears [3]. Bucket-handle tears of the medial meniscus are found three times more frequently than bucket-handle tears of the lateral meniscus [3]. The presence of this type of tear in both the medial and lateral menisci has been reported as a result of either an acute injury or a deficiency of the anterior cruciate ligament [4-6]. We describe a case of acute bucket-handle tear of the medial and lateral menisci with intact anterior and posterior cruciate ligaments. To the best of our knowledge, there are no previous reports in the literature of this kind of injury.

## Case presentation

A 28-year-old Caucasian Greek woman presented to the emergency department after sustaining an injury to her right knee during dancing. The mechanism of her injury compromised vigorous internal rotation of the femur on the tibia with the knee in flexion. She complained of mild pain in both the medial and lateral aspects of her knee joint. Her right knee was locked in 35° of flexion.

**Figure 1:**
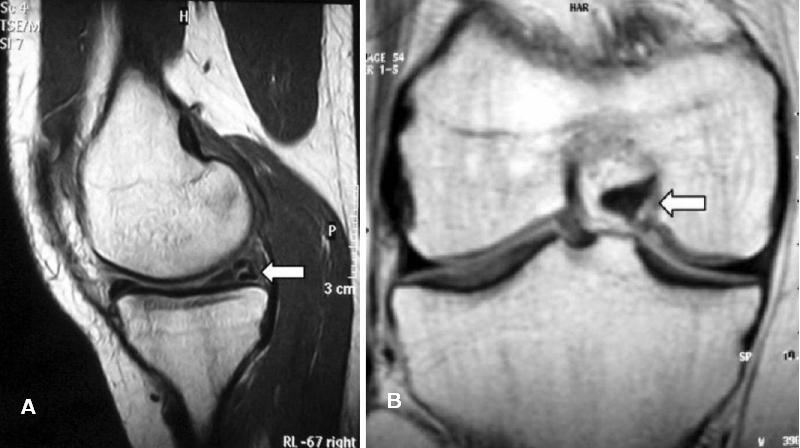
**(A) Sagittal and (B) coronal magnetic resonance views revealed tears of both menisci of the right knee (arrows)**.

**Figure 2:**
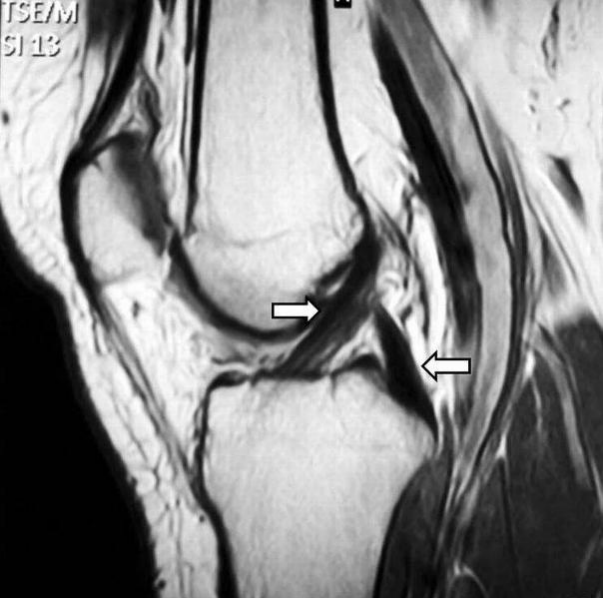
**Magnetic resonance imaging evaluation demonstrated normal signal of both anterior and posterior cruciate ligaments (arrows)**.

Physical examination demonstrated negative Lachman-Noulis and anterior drawer tests. Clicks or catches were not detected by palpation during flexion, extension, and rotary motions of her knee joint. There was tenderness in her medial and lateral joint line. Results of standard anteroposterior and lateral roentgenograms were normal. A magnetic resonance imaging (MRI) evaluation demonstrated the tears in both menisci of the right knee (Figure 1), while the anterior and posterior cruciate ligaments were found to be intact (Figure 2).

Our patient had no relevant medical history. Her physical examination and laboratory tests were unremarkable. No diseases of the connective tissue or other deficiency of the ligaments were detected.

**Figure 3:**
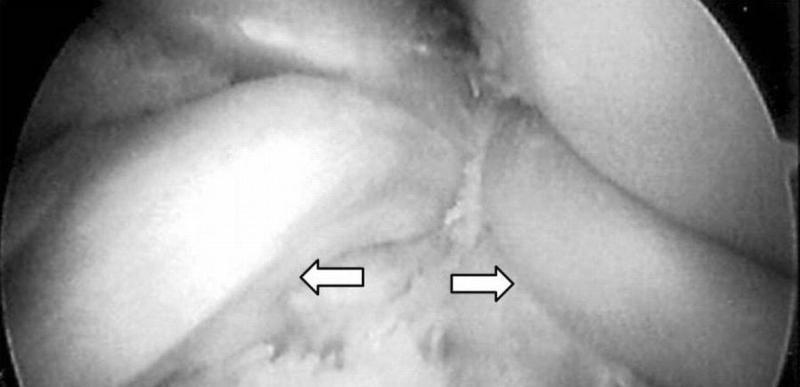
**Arthroscopic appearance of bicompartmental bucket-handle tears (arrows)**.

She underwent a knee arthroscopy in the next 24 hours, and the diagnosis of combined injury of bicompartmental bucket-handle tears with an intact anterior cruciate ligament was confirmed (Figure 3). Arthroscopic evaluation also revealed a discoid lateral meniscus. Moreover, a grade III chondral lesion (graded on the Outerbridge classification), less than 1 cm^2 ^in size, was detected on the medial femoral condyle [7]. A partial medial and lateral meniscectomy was then performed. The decision was made because of the discoid lateral meniscus and the quality of the ruptured part of the medial meniscus. The torn tissue - approximately 40% of the mediolateral width of each meniscus - was removed. The cartilage defect was repaired by drilling therapeutic holes (approximately 0.5 to 1.0 mm in diameter) into the subchondral bone marrow space underlying the region of the cartilage lesion (microfracture technique) [8].

At our patient's follow-up examination six months after her injury, clinical tests demonstrated that her right knee was stable, had a full range of motion and had no tenderness. The functional outcome was evaluated using Lysholm scoring. At the time of her presentation to our emergency department she had a Lysholm score of 75, while six months after her injury, her Lysholm score had increased to 85 [9]. She was satisfied with the outcome of the operation and returned to her pre-injury activities.

## Discussion

Bucket-handle is a type of meniscal tear that extends from the posterior horn to the anterior horn when the inner fragment is displaced into the intercondylar notch. Most studies describe bucket-handle tears as comprising approximately 10% of all meniscal tears. Some authors have reported a higher incidence, with the bucket-handle tear occurring in even 26% of menisci injuries [10,11]. The injury usually occurs in young and middle-aged adults, resulting from an acute injury to the knee joint.

Bucket-handle tears are most common among soccer players. It is generally believed that there is no specific mechanism of injury that could lead to this type of meniscal lesion. The fact that there are cases in which bucket handle tears occur without any history of injury to the knee joint, as in our patient, gives rise to theories that bucket-handle tears may be due to degenerative changes in the menisci [3].

Meister *et al. *suggested that tears in the avascular zone of the medial meniscus that occur in the presence of an intact anterior cruciate ligament may be secondary to a pre-existing, ongoing and underlying disease process [12]. Correlating the arthroscopic image of the torn menisci, the age, the overall level of activity, the medical history and the mechanism of injury in our patient, we conclude that the tear of both the medial and lateral menisci should be considered as the outcome of pre-existing and underlying degenerative changes in the menisci, rather than as the result of a specific mechanism of injury. A previous injury of the knee joint could signal the onset of minor degenerative changes in the meniscus during the healing process. These changes were not significant enough to cause symptoms during the 18-month interval between the two injuries, but they could be a risk factor for the bucket-handle tear in both menisci after the impact of the second injury.

Bucket-handle tears are usually found in the medial meniscus. According to some authors, patients with acute injury of the knee joint and consequent anterior cruciate ligament deficiency are more likely to sustain a bucket-handle tear in the medial meniscus than in the lateral meniscus [13]. The reported incidence of bicompartmental locked bucket handle tear with anterior cruciate ligament injury varies from 7% to 20% [14,15]. To the best of our knowledge, there are only three cases describing a combined acute injury of both the medial and lateral menisci with concomitant anterior cruciate ligament injury [4-6].

Although the common presenting symptom of a patient with a bucket-handle tear is locking of the knee joint, no history of locking occurred in 20% of reported cases [3]. Symptomatology may also include pain, poor joint mobility, edema and hematoma. However, these symptoms are not pathognomonic for bucket-handle tears.

The most important diagnostic tool for the confirmation of the clinical suspicion of a bucket-handle tear is MRI. Six different MRI signs have been described in the literature for bucket-handle tears: (i) fragment within the intercondylar notch sign; (ii) absence of the bow tie sign; (iii) disproportional posterior horn sign; (iv) double posterior cruciate ligament sign; (v) double anterior horn sign; and (vi) flipped meniscus sign. The overall sensitivity to MRI of bucket-handle tears range from 45% to 98%, with some signs having a specificity of 100%. A discoid meniscus which has a thickened body portion, can have the normal bow tie appearance even when a bucket-handle tear is present. In our patient, one of the menisci that sustained a bucket-handle tear was a discoid meniscus.

Thoreux *et al. *[16] demonstrated that MRI plays an important role in predicting the reparability of a tear. Despite the great contribution of MRI, however, arthroscopy remains the gold standard for the diagnosis, offering at the same time suggestions for treatment.

Because of the importance of the meniscus in load transmission across the knee joint, as well as its unique role in load transmission, many studies suggested the need to preserve the maximum area of the injured meniscus. Recently, Shelbourne and Dickens reported favourable radiographic and subjective results after a long term follow-up of 12 years [17]. Although suturing bucket-handle tears remains a viable option, we performed a partial meniscectomy of the affected meniscus in each side.

## Conclusion

Ours is the first case in the literature that describes a combined bucket-handle injury of both the medial and lateral menisci with an intact anterior cruciate ligament. The clinical examination of the anterior cruciate ligament was unremarkable with no signs of deficiency or rupture. The posterior cruciate ligament was also intact. On MRI, the ligaments were visualised as intact in all their length. These findings were confirmed by arthroscopic evaluation.

## Consent

Written informed consent was obtained from the patient for publication of this case report and any accompanying images. A copy of the written consent is available for review by the Editor-in-Chief of this journal.

## Competing interests

The authors declare that they have no competing interests.

## Authors' contributions

CDP, MGL, GIM and NP were involved in the patient care, acquisition of data, analysis and interpretation of data, review of literature, and drafting and revising the manuscript. AEB was involved in review of the literature. He also revised the manuscript for important intellectual content. All authors read and approved the final manuscript.
